# Applying molecular dynamics simulations to validate OCT1 substrates uncovers vitamin K1 as a high-affinity ligand

**DOI:** 10.3389/fphar.2026.1830280

**Published:** 2026-06-19

**Authors:** Elham Omer Mahgoub, Reem Ali, Ayman Samara, Dindial Ramotar

**Affiliations:** 1 College of Arts and Sciences, Qatar University, Doha, Qatar; 2 Division of Biological and Biomedical Sciences, College of Health and Life Sciences, Hamad Bin Khalifa University, Doha, Qatar; 3 Qatar Environment and Energy Research Institute, Hamad Bin Khalifa University, Doha, Qatar

**Keywords:** docking and molecular dynamics simulations, drug–drug interactions, ethidium bromide, metformin, organic cation transporter 1 (OCT1), substrate specificity, transport, vitamin K1

## Abstract

OCT1 is a hepatic membrane uptake transporter of clinical importance in drug metabolism and pharmacokinetics. OCT1 exhibits broad substrate specificity, mediating uptake of diverse compounds, including the neurotransmitter serotonin and the antidiabetic drug metformin. Numerous compounds have been reported as OCT1 substrates using *in vitro* model cell lines overexpressing the transporter and computational prediction approaches, often benchmarked against the prototypical substrate tetraethylammonium. The reported substrate affinities span a wide kinetic range, with *K*_*m*_ values from 0.48 µM for prostaglandin F2α and prostaglandin E2 to 33,900 µM for trimethylamine N-oxide, highlighting the potential for clinically relevant drug–drug competition during cellular uptake. In this study, we establish an integrated framework to evaluate OCT1 substrates using *in silico* docking and molecular dynamics simulations, complemented by *in vitro* transport assays. The computational analysis concurs with previous *in vitro* cell line studies that ethidium bromide is a high-affinity substrate for OCT1, and herein, we validated experimentally that OCT1-deficient HEK293 cells showed negligible uptake of the compound. Competitive uptake experiments further demonstrated that metformin significantly inhibits ethidium bromide transport, indicating that under these conditions, metformin could be a high-affinity OCT1 substrate. In addition, our approach revealed for the first time that vitamin K1 potently inhibits OCT1-mediated transport of ethidium bromide in HEK293 cells. The integration of computational modeling with experimental validation provides a robust strategy for identifying and characterizing high-affinity OCT1 ligands and highlights the importance of OCT1 in drug transport, nutrient uptake, and potential drug–drug interactions.

## Introduction

The poly-specific organic cation transporter OCT1 (SLC22A1) is a member of the SLC22A solute carrier family that includes OCT1, OCT2, OCT3, OCTN1, OCTN2, and OCTN3 (Koepsell et al., 2003). OCT1 is principally expressed in the liver and present on the basolateral membrane of hepatocytes. It can transport numerous endogenous and exogenous compounds, including thiamine, acetylcholine, serotonin, and many drugs, including metformin, morphine, and berberrubine, when compared to a prototypical cationic compound such as 1-methyl-4-phenylpyridinium (Meyer et al., 2020; Grundemann et al., 2005; Brosseau and Ramotar, 2019). In 2021, two independent groups from Germany (Jensen et al., 2021; Haberkorn et al., 2021), reported separate approaches that identified and validated substrates of OCT1, including (i) a machine learning-guided virtual screen, and (ii) compiling data from the literature that exploited the *in vitro* cell line HEK293T transfected with a recombinant plasmid overexpressing the OCT1 transporter to measure intracellular drug uptake (Haberkorn et al., 2021). This OCT1-transfected cell line approach is endorsed by the U.S. Food and Drug Administration (FDA), as well as the European Medicines Agency to test new pharmaceuticals for drug-drug interactions, and it has the advantage that it can be modified to also measure transcellular transport of substrates (Haberkorn et al., 2021). In this *in vitro* cell system, substrate uptake rates and affinities are calculated from the difference between the endogenous OCT1 expression level and that of the recombinantly overexpressed OCT1 (Haberkorn et al., 2021).

Using the above-mentioned *in vitro* cell system for preclinical drug development has many inherent limitations. It can burden the cells with OCT1-overexpressed proteins that overwhelm the protein folding and modification pathways, leading to a significant fraction of non-functional proteins (Marinko et al., 2019). There is also a real concern that improperly folded or modified OCT1 protein may not embed into the plasma membrane, or that overexpression of the protein may displace other solute carriers from the membrane (Marinko et al., 2019). While this *in vitro* cell system offers a simple and convenient approach, it raises many other challenging questions, such as whether the overexpressed OCT1 accurately reflects its substrate specificity or whether it results from a stimulatory effect on other non-specific endogenous solute carriers. More importantly, it is uncertain to what extent overexpression of OCT1 in the uptake system generates fragmented forms of the protein, as antibodies specific to OCT1 are not commercially available to detect the native or the truncated forms (Marinko et al., 2019). Thus, it remains possible that fragments of OCT1 could compete and interfere with the post-translational modifications of the endogenous levels affecting its activity, and likewise, displacing it from the plasma membrane, and this could be problematic, particularly since many human membrane transporters are expressed at relatively low abundance (Prasad et al., 2017).

So far, cell lines deleted for the OCT1 gene have not been used to monitor for the absence of substrate uptake, residual uptake, or the reuptake of the substrate if the gene-deletion cell line is engineered to re-express the OCT1 protein (Brosseau and Ramotar, 2019). Although OCT1 null mice exist, they have not been exploited to test the effects of the broad range of substrates reported to be transported by OCT1 (Jonker et al., 2003). The review by Haberkorn et al. compiled a list of compounds, including several FDA-approved drugs that have been reported as substrates for OCT1, using cells transfected to overexpress OCT1 (Haberkorn et al., 2021; Hendrickx et al., 2013). In another approach, many OCT1-interacting compounds were identified by machine learning, which used several parameters, including the spatial relation of chemical and molecular structures, to virtually screen a database of compounds (Kumar et al., 2025; Jensen et al., 2021). The analysis provided some notable characteristics for the substrates of OCT1, and these include that the substrates possess a volume of ∼500 Å, at least one positive charge, and or one aromatic ring. These reported substrates have measurable *K*_*m*_ values for OCT1, ranging with a *K*_*m*_ value from as low as 0.8 μM for fenoterol, a drug used for treating asthma, to as high as 1470 μM for the drug metformin used for treating type 2 diabetic patients, and even a higher *K*_*m*_ value of 1997 μM for the essential vitamin thiamine (Meyer et al., 2020). Since OCT1 transports many FDA-approved drugs, individuals simultaneously taking more than one FDA-approved drug are likely to face the dilemma of drug-drug competition, such that they do not receive the full benefits from either drug (Kumar et al., 2025). For example, drugs with high affinity, such as the painkiller morphine (with *K*_*m*_ value of 3.4 μM) and the polyphenol antioxidant quercetin (with *K*_*m*_ value of 2.2 μM), will outcompete those prescribed drugs with lower affinities, such as metformin, when taken together and assuming that the calculated *in vitro K*_*m*_ values are close to the *in vivo K*_*m*_ values. At least 40%–67% of all drugs are classified as organic cations, and it seems important to implement additional tools to better assess the competition of drugs into cells via the OCT1 transporter (Koepsell, 2020).

In this investigation, we used docking and molecular dynamics simulation methods to forecast the binding energy and interaction of OCT1 with all substrates mentioned by Jensen et al. (2021) and Haberkorn et al. (2021) to assess if the predicted energy values corresponded to the reported substrate affinities for the OCT1 transporter. The resulting interactions demonstrate that not all substrates with high affinity have the lowest binding energy to OCT1. This difference underscores the importance of other factors, such as solvation and kinetic effects, as well as the transporter conformational flexibility (Stewart, 2025; Aghajani et al., 2022). It suggests that binding affinity cannot be explained solely by energy values but rather requires a more comprehensive understanding of transporter-substrate interactions (Alonso et al., 2006).

Molecular dynamics analyses identified energetically favorable and persistent ligand–OCT1 interactions, including those with metformin and vitamin K1, suggesting that these compounds could competitively occupy the OCT1 transport pathway. As such, we assessed whether these ligands interfere with OCT1-mediated uptake of the substrate ethidium bromide, a fluorescent compound defined as a high-affinity substrate for OCT1 (Lee et al., 2009; Mihaljević et al., 2017). We showed that ethidium bromide at very low concentration entered the cells via the OCT1 transporter, and very weakly into the HEK293-OCT deleted cells. Interestingly, metformin was effective in competing for ethidium bromide uptake at the same initial drug concentrations. This latter observation is inconsistent with the reported higher *K*_*m*_ value (1470 μM) of metformin vs. the lower *K*_*m*_ value (0.8 μM) of ethidium for the OCT1 transporter (Lee et al., 2009; Mihaljević et al., 2017; Kimura et al., 2005), suggesting that more than one tool is needed to assess whether a compound is a valuable substrate for the transporter. We further explore the tools described herein to test whether other compounds could be suitable substrates for OCT1.

## Materials and methods

### Ligand design and docking

Two independent datasets of OCT1 substrates were analyzed in this study. 138 compounds of OCT1 experimental substrates were extracted from Haberkorn et al. (2021) while an additional 45 predicted substrates were included from Jensen et al. (2021), Haberkorn et al. (2021). The two studies employed different approaches to validate OCT1 substrates. Haberkorn et al. validated OCT1 substrates experimentally using a single-transfected cell line recombinantly overexpressing OCT1, while Jensen et al. applied a machine learning-based approach to predict potential OCT1 substrates. Compounds identified from both studies were included in the present work for molecular docking analysis. The compounds were drawn as 2D structures using ACD/ChemSketch software and saved as MDL mol files. The structures were subsequently converted to PDB format using OpenBabel GUI software for use as ligand input files in docking studies. Preparation of both the ligand and OCT1 protein files was performed using AutoDock, which was also used to define the active-site coordinates (grid box). Docking was executed in the Cygwin64 terminal environment. Ligands exhibiting the lowest binding energy scores were considered for further analysis. Based on the selected binding energy cutoff, 22 compounds were retained. In addition, Vitamin K1, Menadione, and Olaparib were included for comparative evaluation. Collectively, these 25 compounds were subsequently subjected to ligand filtration analysis.

### Apo-OCT1 transporter MD simulation

The structure of the OCT1 protein was obtained from the Protein Data Bank using PDB I 8sc1. Molecular dynamics (MD) simulations were performed using GROMACS with the CHARMM36 force field. Protein topology files were created using the pdb2gmx module. The protein was solvated using the simple point charge (SPC) water model in a simulation box with periodic boundary conditions, and appropriate counterions were added to neutralize the system charge. Energy minimization was performed using the steepest descent algorithm for up to 10,000 steps, until the maximum force was below 1000 kJ/mol/nm, to remove steric clashes and unfavorable contacts. System equilibration was subsequently performed in two phases: an NVT (number of particles (N), volume (V), and temperature (T) remain constant) equilibration step for 50,000 steps to stabilize the system temperature, followed by an NPT (number of particles, pressure (P), and temperature remain constant) equilibration step for 100,000 steps to stabilize system pressure and density.

Following equilibration, a production MD simulation was conducted for 1 ns to evaluate the structural stability of OCT1. Post-simulation trajectory analyses, including root mean square fluctuation (RMSF) and root mean square deviation (RMSD) were performed using GROMACS analysis tools.

### Ligand preparation for molecular dynamics simulations

The simulations were conducted in an aqueous environment without explicit inclusion of a lipid bilayer This simplified setup was chosen to focus on ligand–binding interactions within the transporter cavity. The simulations used the water model and GROMACS version 4.0 (Win64-multicore), which incorporated the CHARMM 36 force field. GROMACS pdb2gmx modules were used to generate protein and ligand topologies. The number of ligands required to get MD simulation is reduced through topology filtration. Each ligand topology was validated by checking missing parameters, charge consistency, and structural integrity using GROMACS tools: Ligand structures were initially obtained in PDB format, from docking outputs. These were visualized and cleaned using PyMOL software, where alternate conformations were removed. Second, hydrogen atoms were added to clean PDB files after they were imported into the Avogadro software. The structures were subsequently saved in MOL2 format, which preserves atom types and partial charges, after being energy-minimized using the MMFF94 force field. Then the CGenFF (CHARMM General Force Field) server received the MOL2 ligands files and the matching’s topology files were produced. These files include partial charges, bonded and non-bonded parameters, and atom types that work with simulations that use CHARMM. Later the topology Integration for The cgenff_charmm2gmx.py script were parses CHARMM parameters which then was used to convert the. str files into the file. itp (include topology), file. prm (parameter) and files. top (ligand topology) which they all coordinate GROMACS-compatible forms. File. gro that used in further production of file. Complex.

### Molecular dynamics simulations

The OCT1 protein topology and the ligand topology were combined using pdb2gmx in the GROMACS module to enable file Complete system assembly. After neutralizing the system with water models (usually TIP3P) and counterions, energy minimization and equilibration procedures were performed. The NVT equilibration step of the molecular dynamics workflow involves stabilizing the system for 100 ps at 300.5 K while maintaining constant NVT conditions. The switch to NPT equilibration was then made possible by updating the topology file to include the ligand parameters. The system was equilibrated under constant NPT conditions in the second phase, ensuring thermodynamic stability. Position restrictions were removed after equilibration was finished, and a 1-ns production MD simulation started to evaluate the complex’s stability. After that, structural studies were carried out, In a Linux-based GROMACS workflow, the four core MD simulation parameters can be analyzed with the following commands: RMSF (Root Mean Square Fluctuation**)** is computed using **gmx rmsf -s topol. tpr -f traj. xtc -o rmsf. xvg -res** to evaluate residue flexibility; RMSD (Root Mean Square Deviation) is obtained with **gmx rms -s topol. tpr -f traj. xtc -o rmsd. xvg -tu ns** to monitor structural stability relative to a reference; **Hydrogen Bonding** analysis is carried out via **gmx hbond -s topol. tpr -f traj. xtc -num hbnum. xvg -hbn hbond. ndx -hbm hbond_map.xpm** to quantify hydrogen bond numbers and lifetimes; **Radius of Gyration (Rg)** is determined with **gmx gyrate -s topol. tpr -f traj. xtc -o gyrate. xvg** to assess molecular compactness; and **Free Energy (ΔG)** can be extracted through umbrella sampling using **gmx wham -it tpr-files. dat -if pullf-files. dat -o profile. xvg,** which generates the potential of mean force along a chosen reaction coordinate.

### Cell culture and SLC22A1 (OCT1) deletion by CRISPR Cas9

HEK293T cell lines (American Type Culture Collection, USA) were maintained in Dulbecco’s modified Eagle’s medium (DMEM) with 10% fetal bovine serum (FBS) and 1% Penicillin streptomycin. OCT1 knock-out by CRISPR cas9 were performed following the protocol described previously (Joung et al., 2017). Briefly, gRNA oligos targeting OCT1 were cloned into the px330 plasmid vector gifted by Feng Zhang (Addgene plasmid # 42230). Cloning was verified by Sanger sequencing. Transfection of plasmids was performed in HEK293T using Fugene HD transfection reagent. Putative clones with the modified OCT1 gene were verified by Sanger DNA sequencing.

### RT-PCR analysis

mRNA was isolated from HEK293 and HEK293_OCT1_KO cells using the RNeasy mini kit (Qiagen) as per the supplier’s protocol. RNA was quantified using a NanoDrop (Thermo Fisher), and 2 µg of RNA was transcribed to cDNA using the High-Capacity cDNA Synthesis Kit (Applied Biosystems) according to the manufacturer’s protocol. Real-time PCR (qPCR) was performed using SYBR Green master mix (Thermofisher) on the Quanti Studio 6 Flex machine. GADPH was used as a housekeeping gene. The primers sequence used were as follows: GADPH-F, 5′-GTC​TCC​TCT​GAC​TTC​AAC​AGC​G; GADPH-R, 5′-ACC​ACC​CTG​TTG​CTG​TAG​CCA​A, OCT1 -F, 5′-CGC​ACC​TTC​ATC​CTG​ATG​TAC​C; and OCT1-R, 5′-GAG​CGG​AGT​AAA​GGA​AAT​CCA​GG.

### Clonogenic survival assay

HEK293T control and HEK293T OCT1 KO cells were plated in 6-well plates overnight at ∼ 250 cells/well density. The next day, cells were challenged with compounds (Olaparib, Menadione, or Metformin) at the indicated concentrations for 1 h. The media were changed to fresh media, and the cells were incubated for 10 days to form colonies. After incubation, colonies were stained with crystal violet in an acetic acid-methanol mixture. For the Menadione and metformin competition experiment, cells were treated with the indicated doses of Menadione plus 10 µM Metformin or 20 µM Metformin and incubated for 1 hr. Then the media were changed, and the cells were left to form colonies for 10 days.

### Confocal microscopy

HEK293T control and HEK293 OCT1 KO cells were seeded on coverslips pre-coated with poly-D-lysine. Cells were treated with ethidium bromide (10 μm for 3 min). The cells were fixed in 4% paraformaldehyde for 20 min, and nuclei were stained with DAPI for 1 hr. Imaging was performed using a Leica SP2 confocal Laser scanning microscope.

### Equation used for the radius of gyration ( $R_{g}$ )

The following equation is commonly used in molecular dynamics, structural biology, and protein folding studies to measure how compact or spread out a molecule is around its center of mass. When the $R_{g}$ is low, the molecule is compact or tightly folded, and when it is high, the molecule is unfolded. The equation was used during the molecular dynamics simulations of OCT1 to determine structural stability, ligand-induced conformational changes, and whether the protein becomes more compact during simulation. In the equation, $m_{i}$ is the mass of an atom i , $r_{i}$ is the position of the atom i , and $r_{cm}$ is the center of mass of the whole molecule. The equation calculates the mass-weighted average distance of all atoms from the center of mass of the molecule, and stable $R_{g}$ over the time scale means the protein remains structurally stable.

$$R_{g}=\sqrt{\frac{\sum_{i}m_{i}{\left(r_{i}-r_{cm}\right)}^{2}}{\sum_{i}m_{i}}}$$

## Results

### Comparison of OCT1 substrate binding energies with reported *K*_*m*_ values reveals little correlation

**FIGURE 1:**
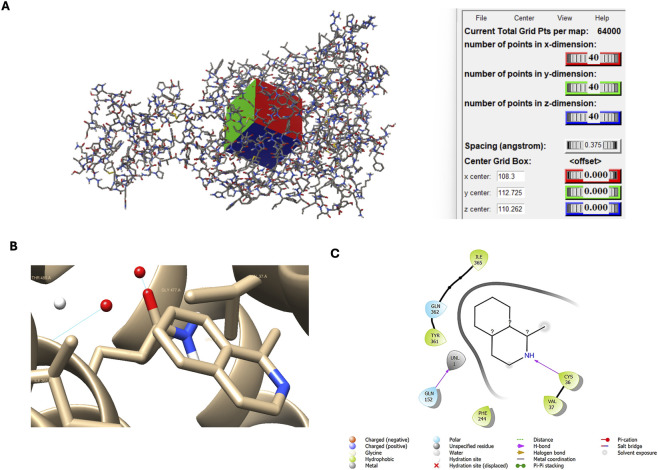
Docking representation of OCT1 and its interacting substrates. **(A)** The OCT1 transporter interior binding cavity was covered by the docking grid box, which was centered at x = 108.3 °, y = 112.725 °, and z = 110.262 °. All compounds were consistently docked into the inward-facing model using this configuration grid box in Autodock. **(B)** 3D structural visualization of OCT1 bound to salbutamol, with hydrogen bonds displayed in light blue lines generated by UCSF-Chimera software. The salbutamol was positioned centrally within the binding pocket. **(C)** 2D structural representation of OCT1–salbutamol interactions, with hydrogen bonds indicated by the two pink arrows. Active amino acids surrounding the binding pocket are shown in distinct colors to display their roles in the substrate interactions using Maestro software. The color key is shown in Figure 1C. UNL refers to the unlabelled ligand identifier used in the structural file.

Many transporters have a defined range of substrates, and OCT1 appears to be a promiscuous transporter with numerous substrates (Jensen et al., 2021; Haberkorn et al., 2021; Zeng et al., 2023). As such, we first checked whether the published substrates could be validated by ligand-interaction to the OCT1 transporter (see Materials and Methods). We analyzed two separate lists of substrates, one compiled by Haberkorn et al. (2021) with substrates reported over the last two decades, and another by Jensen et al. (2021) that were deduced by machine learning tools (Haberkorn et al., 2021). Both lists contained common and unique substrates that were subjected to docking analysis. Briefly, docking studies were performed using AutoDock and Cygwin64 Terminal, yielding 138 compounds that docked to OCT1 (Supplementary Table S1). Figure 1A shows the inward-facing conformation of OCT1 (PDB ID: 8sc1), selected because it represents the substrate-release state towards the cytoplasm, which is physiologically relevant for evaluating ligand interactions within the transporter cavity. The docking grid box, centered at x = 108.3 °, y = 112.725 °, and z = 110.262 °, covered the inner binding cavity of the OCT1 transporter. All compounds identified by docking were consistently docked into an inward-facing orientation, and many cationic substrates were reported to exhibit stable interactions within the inward-facing cavity, for example, metformin (Zeng et al., 2023).

We set a binding energy cutoff between −6.8 and −2.86, yielding 21 compounds of the 138 (Table 1). We included metformin as a well-studied substrate, but with a lower binding energy of −1.95 kcal/mol, bringing the total to 22 (Table 1). Most of the selected substrates that docked onto OCT1 have corresponding reported *K*_*m*_ values (Table 1) (Jensen et al., 2021). We also included 5 compounds (for example, nitidine and quercetin) that do not interact with OCT1, although these have been shown to be taken up by the transporter as indicated by the reported *K*_*m*_ values (Table 1) (Haberkorn et al., 2021). In addition, we included 3 potential substrates that have not been previously shown to interact with OCT1 (Table 1).

**TABLE 1:** Comparing substrate binding energy with their published *K*_*m*_ value.

Compounds	Binding energy (kcal/mol)	Calculated. *K*_*m*_ values (µM)	Hydrogen bond	Amino acid residues	Estimated charge at pH 7.4
Docked substrates with low binding energy					
1-(m-phenoxyphenyl)-biguanide	-6.8	ND	none	Gln _362_, Cys _36_, Phe _159_	1
1-(p-chlorophenethyl)-biguanide	-6.47	ND	none	Cys _473_, Gln _362_, Ser _358_, Ile _446_	1
2,3-dihydro-1H-inden-2-yl-biguanide	-5.91	ND	none	Leu _148_, Gyn _43_, Val _40,_ Ile _39_, Leu _364_, Gly _369_, Ala _270_	1
Edrophonium	-4.19	26.4 + 9.1	two	Gln_152_, Gln_362_, Cys_36_, Val_37_, Ile_365_	1
Reproterol	-4.19	ND	one	Gln_152_, Gln_362_, Cys_36_, Val_37_, Ile_365_, Phe_244_	1
Ethidium	-3.01	0.8 + 0.2	one	Gln_152_, Gln_362_, Cys_36_, Val_37_, Ile_365_,	1
Etilefrine-(S)	-4.83	35.7 + 7.3	one	Gln_152_, Gln_362_, Cys_36_, Val_37_, Ile_365_	1
Noroxycodone	-3.85	20.05 ± 6.5	one	Gln_152_, Gln_362_, Cys_36_, Val_37_, Ile_365_	1
Phenformin	-4.82	ND	one	Gln _362_, Ile _365_, Gln _152_, Val_37_, Cys _36_	1
Salbutamol-(R)	-5.03	224.2 ± 18.4	two	Ile _365_, Gln _362_, Tyr _361_, Gln _152_, Phe _248_, Val _27_, Cys _36_	1
Sparteine	-3.94	27.2 ± 2.8	one	Val_17_, Cys _36_, Asn _158_, Ile _263_, Gln _362_, Tyr _361_, Gln _132_	1
Tropisetron	-4.04	ND	one	Tyr _361_, Gln _362,_ Val _37_, Cys _36_, Phe _244_	1
Xamoterol (R)	-3.94	ND	two	Gln _363_, Tyr _361_, Cys _473_, Ser _358_, Cys _36_	1
YM155	-3.22	22.1 ± 2.5	none	Gln_152_, Gln_362_, Cys_36_, Val_37_, Ile_365_, Phe_159_	1
Ganciclovir	-5.93	516.2 ± 70.3	none	Ile_39_, Gln_152_, Cys_36_, Gln_362_, Ile_365_, Leu_366_	Neutral
Lamivudine	-3.45	249 ± 51	none	Gln_152_, Cys_36_, Leu_308_, Gln_362_, Ile_39_, Val_27_	Neutral
Serotonin	-5.40	197 ± 42	none	Gly_427_, Gln_152,_ Asn_136_, Thr_469_, Pro_481_	1
Morphine	- 4.15	3.4 ± 0.3	none	Gln_152_, Cys_36_, Val_37_, Gln_362_, Tyr_361_	1
Thiamine	-4.78	1997 ± 174	none	Gln _152_, Gln _362_, Cys _36_, Val _37_, Ile _365_, Leu_366_, Ile _39_, Val _40_	1
Famotidine	-6.05	35.7 ± 7.3	none	Gln _362_, Tyr _361_, Cys _36_, Val _37_, Ile _365_,	1
Berberrubine	- 2.86	1.27 ± 0.23	none	Ile _265_, Gln _152_, Gln _362,_ Cys _36_, Tyr _381_, Ser _356_	1
Metformin	-1.95	1470 ± 190	one	Gly _427_, Gln _152_, Asn_136_, Thr _469_, Pro _481_	1
Comparison of substrates that do not interact with OCT1					
Nitidine	No interaction	0.797 ± 0.17	​	​	Neutral
Norphenylephrine	No interaction	994 ± 316.5	​	​	Neutral
Oxaloplatin	No interaction	ND	​	​	Neutral
Sorafenib	No interaction	3.8	​	​	Neutral
Quercetin	No interaction	2.2 ± 0.2	​	​	Neutral
Potential substrates not in the list					
Vitamin K1	-6.05	ND	one	Leu _266_, Ile _365_, Phe _249_, Gln _261_, Tyr _263_, Val _37_, Cys _265_, Gln _152_	Neutral
Menadione	+20.56	ND	​	​	Neutral
Olaparib	-4.72	ND	two	Gln _152_, Pro _481_, Ile _39_, Val _37_, Cys _36_, Phe _32_, Phe _159_, Tyr _361_, Gln _362_, Ile _365,_ Leu _366_	Neutral

ND, not determined. The calculated *K*_*m*_ values (µM) are from Haberkorn et al., 2021.

We observed that substrates with low predicted binding energies, suggesting stronger affinity for OCT1, did not always correspond to low experimental *K*_*m*_ values. For example, ganciclovir showed a relatively low binding energy (−5.93 kcal/mol) but was reported to have a high *K*_*m*_ value of 516.2 μM. In contrast, famotidine, with a similar binding energy (−6.05 kcal/mol), exhibited a much lower *K*_*m*_ value of 35.7 μM (Table 1). Interestingly, some compounds reported to have low *K*_*m*_ values (indicating high affinity) for OCT1, such as nitidine and quercetin, did not dock onto the transporter in our analysis (Table 1). These observations indicate that predicted docking binding energies do not consistently correlate with experimentally determined OCT1 substrate affinity.

It is noteworthy that the modeling of the substrates that docked onto OCT1 contacted similar amino acid residues Gln_152_, Gln_362_, Cys_36_, Val_37_, and Ile_365_ (Table 1). Furthermore, if the substrate possesses two or more aromatic rings, additional contacts were made with either Phe_159_ or Phe_244_ or both residues of OCT1 (see Supplementary Table S1; Supplementary Figure S1). The amino acid residues, Gln_152_, Gln_362_, Cys_36_, Val_37_, and Ile_365_, appeared repeatedly in the ligand–OCT1 interaction complexes, raising the possibility that these might be the core residues in OCT1 substrate recognition, and that the Phe_159_ or Phe_244_ residues might be required to recognize the aromatic rings of substrates. The residues are believed to contact the ligands through a mixture of hydrogen bonding (Gln, Cys), hydrophobic packing (Val, Ile), and aromatic interactions (Phe), forming a versatile and conserved interaction surface.

Besides the known compounds, we also separately checked whether other molecules that are stored in the liver, such as vitamin K and its precursor, menadione, would require the function of OCT1 and, as such, dock onto the transporter (Ivanova et al., 2018; Fiore et al., 2001). Additionally, we checked whether the anticancer drug olaparib would dock onto the OCT1 transporter, as it has been inferred to be a potential substrate for OCT1 (Zhao et al., 2023). The computational analysis revealed that vitamin K1 and olaparib, but not menadione, have low binding energies for OCT1, -6.05 and −4.72 kcal/mol, respectively, which were lower than that for metformin (−1.95 kcal/mol), a known substrate for the transporter (Table 1). This analysis suggests that vitamin K1 and olaparib are likely potential interacting ligands of OCT1.

### Hydrogen bonds as interaction energy during docking

Several compounds formed hydrogen bonds with OCT1, which contribute to the predicted interaction energy, as reported in previous studies and indicated in Table 1, and from the 2D structures of docking interaction, see Supplementary Figure S1 (Päslack et al., 2021; Alonso et al., 2006). Docking analysis identifies hydrogen bonds because it uses a static, geometry-based model in which the transporter structure is rigid and favorable interactions are scored, as shown for OCT1 docked with Salbutamol-(R) compound (Figures 1B,C). In contrast, molecular dynamics (MD) simulations provide a dynamic, time-dependent view of the system in the presence of solvent (Alonso et al., 2006; Mahgoub and Kulkarni, 2023). In MD simulations, protein flexibility, water molecules, and thermal motion can disrupt hydrogen bonds predicted by docking. As a result, some compounds that appear to form hydrogen bonds in docking may not maintain these interactions during MD simulations (Vander Meersche et al., 2024).

### Ligand selection for MD simulation analysis

Of the 22 compounds listed in Table 1, we subjected all to ligand topology filter analysis (Supplementary Figure S1), which involved several key stages (see Methods). Initially, the substrate structure in PDB format was exported from PyMol, then imported into Avogadro, where hydrogen atoms were added, and the output file was saved in MOL2 format (Hanwell et al., 2012). This MOL2 file was then submitted to the CGenFF server, which generated the corresponding. str file used to construct the lig. gro file using pdb2gmx in the Gromacs module, as described in the Methods. These filtering steps resulted in eight ligands (Table 2), and Supplementary Figure S2 shows an example of a compound that did not pass the ligand topology filter. Two of the eight ligands (i.e., vitamin K1 and olaparib) that passed the filtering steps were not previously known to be OCT1 ligands.

**TABLE 2:** The eight selected ligand OCT1 complexes.

Ligand	Avg. Radius of gyration (nm)	Max RMSF (nm) with OCT1	Max RMSD (nm)	Averaged ΔG (kcal/mol)	Avg. H-bonds (rounded)	Max RMSF of ligand (nm)
1-(m-phenoxyphenyl)-biguanide_OCT1	2.56	0.45	2	140	2	0.26
1-(p-chlorophenethyl)-biguanide_OCT1	2.48	0.42	1.8	125	1	0.22
2,3-Dihydro-1H-indene-2-yl acetate_OCT1	2.42	0.25	0.4	110	0	0.2
Ethidium_OCT1	2.4	0.22	0.3	95	0	0.35
Metformin_OCT1	2.38	0.2	0.3	85	1	0.18
Sparteine_OCT1	2.36	0.19	0.2	75	1	0.14
Olaparib_OCT1	2.44	0.18	0.2	100	0	0.16
Vitamin K1_OCT1	2.34	0.17	0.2	60	0	0.12

### MD simulation reveals the flexibility of OCT1 by RMSF and RMSD analyses

**FIGURE 2:**
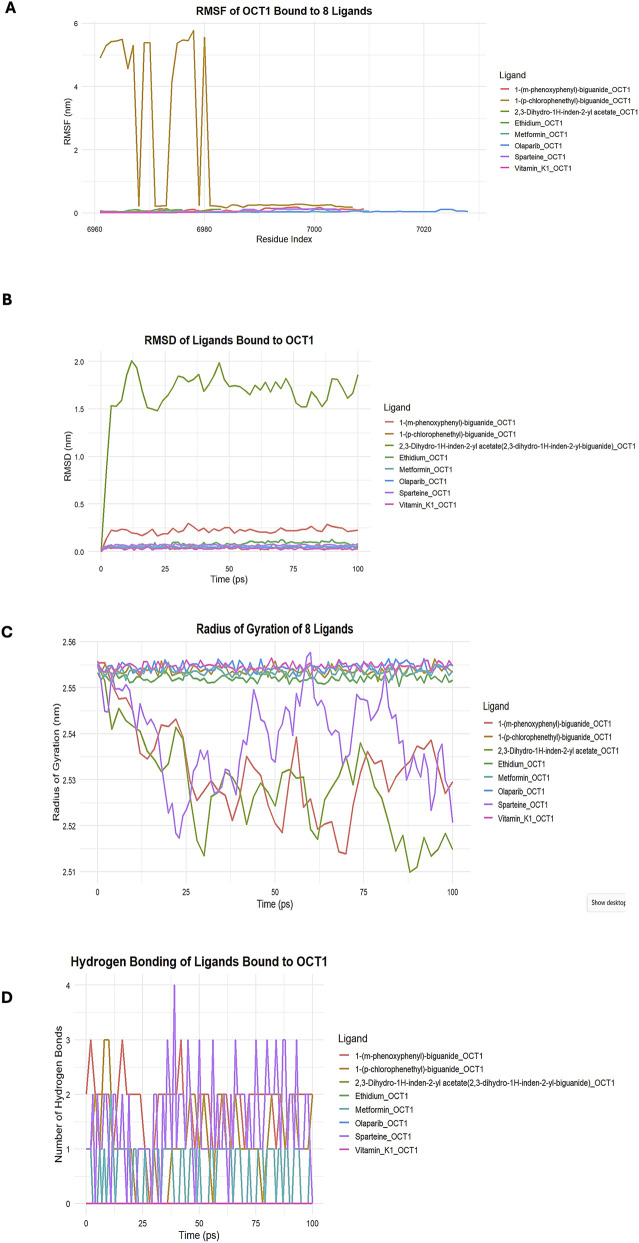
Flexibility and stability of the selected OCT1 ligands. **(A)** The RMSF was determined by molecular dynamics trajectory analysis of OCT1-ligand complexes, indicating areas of stability and flexibility during binding interactions. **(B)** Showing the stability of the ligands connected to the OCT1 protein, and determined using molecular dynamics trajectory analysis based on their root mean square deviation (RMSD, nm) profiles over 100 picoseconds. **(C)** Showing the radius of gyration profiles for the eight ligands bound to OCT1 show their structural compactness, offering information on conformational stability and possible variations in ligand flexibility during binding. **(D)** Hydrogen bond analysis showing four of the eight chosen ligands that establish stronger and longer-lasting polar hydrogen bonds with the OCT1 protein throughout the contacts.

We performed MD simulation analysis of the eight compounds using GROMACS, and the data revealed the RMSF values, as shown in Figure 2A and Supplementary Figure S3A (Materials and Methods). The residue index (the numerical identifier assigned to each amino acid residue around the protein structure) indicated that ethidium was the most flexible across its atomic structure, as compared to the seven other compounds (Supplementary Figure S3A). Five of the seven compounds showed similar flexibility, while two complexes (1-(p-chlorophenethyl)-biguanide_OCT1 and 1-(m-phenoxyphenyl)-biguanide_OCT1) were not flexible (Supplementary Figure S3A). The data also indicated that the two tested molecules, vitamin K1 and olaparib, exhibited nearly the same flexibility as the known substrate metformin (Supplementary Figure S3A), suggesting that both vitamin K1 and Olaparib are likely suitable ligands for OCT1.

The RMSF data also revealed the flexibility of the OCT1 residues around the ligands (Figure 2A). Seven of the eight ligands OCT1 complexes showed very little fluctuations around the OCT1 residue index, while 1-(p-chlorophenethyl)-biguanide showed enormous fluctuations around the 6960–6980 residue index, indicative of the ligand inability to form contact with the protein (Figure 2A). The 6960–6980 residue index corresponded to amino acid residues 230 to 245 of OCT1, which constitutes transmembrane domain 5 (TM5) and it is part of the substrate translocation pathway implicated in substrate recognition and transport (Zhang et al., 2024). The TM5 domain participates in conformational changes during the alternating-access transport cycle (Yee et al., 2024). Together, these plots offer a dual perspective on the molecular motion of the stability of the ligands-OCT1 atoms and residues.

### RMSD analysis reveals stable binding behavior of OCT1-ligand complexes

We next monitored the stability of the ligands bound to the OCT1 protein over a 100-picosecond simulation (Figure 2B). Each colored line represents a different ligand, with RMSD values (in nanometers) plotted against time (ps). Most ligands (ethidium, metformin, olaparib, and sparteine) showed low and stable RMSD profiles (0.05 nm), while it was slightly higher for vitamin K1 (0.25 nm). All the ligands remained stable throughout the simulation, indicating consistent binding behavior as they stabilized around a fixed value and thus formed strong interactions with OCT1. However, **1-(p-chlorophenethyl)-biguanide** showed the highest RMSD deviation between 1.5 and 2.0 nm, indicating less stable binding and weaker interactions with OCT1, consistent with the RMSF data (Figure 2A). This comparative trend highlights the differential conformational dynamics and potential binding affinities among the ligand set and supports the notion that vitamin K1 and Olaparib are likely suitable ligands of OCT1. RMSF measures the average positional fluctuation of individual residues, indicating local flexibility, whereas RMSD records the protein-ligand complex’s overall deviation from its initial structure (Aghajani et al., 2022). A ligand with low RMSF but high RMSD stabilizes local residues while allowing for overall conformational drift, whereas a ligand with high RMSF but low RMSD maintains overall structural stability despite local flexibility (da Fonseca et al., 2024). This explains why chlorophenethyl was stable in RMSD but unstable in RMSF, while 2,3-dihydro showed the opposite behavior. RMSF and RMSD give complementary information about ligand binding stability at both the local and overall conformational levels.

In control analysis, we calculated RMSF and RMSD for the apo form of OCT1 and compared them with those of the OCT1-ligand-bound complexes to evaluate the stabilizing effect. Due to the essential flexibility of the transporter in its unbound state, the apo-OCT1 showed RMSF values in the range of ∼0.40 to 0.65 nm (Supplementary Figure S3B), while it displayed moderate backbone deviations, with RMSD values changing between 0.40 and 0.65 nm throughout the simulation (Supplementary Figure S4). Conversely, most of the ligand-bound complexes had lower RMSF values (<1.0 nm) (Figure 2A), showing residue-level stabilization, and lower RMSD values (<0.5 nm) (Figure 2B), indicating improved overall stability. The 2,3-dihydro-1H-inden-2-yl acetate derivative deviated significantly from this pattern (Figure 2B), exhibiting RMSD values of ∼2.0 nm, consistent with OCT1 instability relative to the apo baseline (Supplementary Figures S3, S4).

### Structure compactness during MD simulation reveals ligand stability with OCT1

We next determined the radius of gyration (y-axis, in nanometers) over simulation time (x-axis, in picoseconds) for the eight ligands bound to OCT1. The results are shown by a line graph displaying the structural compactness of the ligand-OCT1 during the molecular dynamics simulations (Figure 2C). Five of the ligands, including Olaparib and vitamin K1, exhibited relatively stable structures across the 100 ps window, indicating consistent conformational behavior (Figure 2C). However, sparteine, 2,3-Dihydro-1H-indene-2-yl acetate_OCT1, and 1-(m-phenoxyphenyl)-biguanide_OCT1 showed noticeable fluctuations, suggesting dynamic structural changes. In reference to Figure 2C and Table 2, the ligands, including vitamin K1_OCT1 (2.34 nm) and olaparib (2.44 nm), maintained lower average radius of gyration values (0.2 nm), implying compact and potentially more stable conformations. The radius of gyration is defined mathematically as the mass-weighted root mean square distance of atoms from the center of mass (see Materials and Methods). The average *Rg* values, as (2.56–2.34 nm), represent the time-averaged compactness of each ligand–protein complex. Conversely, 1-(m-phenoxyphenyl)-biguanide_OCT1, with the highest average value of 2.56 nm, appeared more expanded, which may reflect increased flexibility or lower binding stability (Figure 2C). This metric provides valuable insight into ligand behavior within the OCT1 binding pocket.

### Molecular dynamics simulation reveals different energy bonding of ligands with OCT1

Among the eight ligands bound to OCT1, sparteine exhibited the highest average number of hydrogen bonds (between three and 4), suggesting stronger and more sustained polar interactions. Three of the ligands, 1-(m-phenoxyphenyl)-biguanide, 1-(p-chlorophenethyl)-biguanide, and metformin have between, 3 to 2, 2 to 3, and one to two hydrogen bonds, respectively, indicating metformin has a moderate bonding stability (Figure 2D). Surprisingly, 2,3-dihydro-1H-inden-2-yl acetate, ethidium, olaparib, and vitamin K1 in complex with OCT1 showed no hydrogen bonding, implying minimal contribution of hydrogen bonds to their binding affinity. The analysis indicates that although some compounds form hydrogen bonds with OCT1, others do not, suggesting that these substrates may rely on other interactions (e.g., hydrophobic interactions) to form stable structures.

We performed a Molecular Mechanics Poisson–Boltzmann Surface Area (MM-PBSA) calculations and the energy breakdown revealed that ethidium_OCT1 possessed the strongest binding affinity (−33.4 kcal/mol), driven by excellent van der Waals and gas-phase interactions. Sparteine_OCT1 also showed strong binding (−31.7), aligning with its favorable MD and Km profile. The biguanide derivatives, olaparib_OCT1, and vitamin K1 exhibited similar binding energies (−29 kcal/mol). In addition, the ligands displayed similar van der Waals, electrostatic, and solvation energies (Table 3), suggesting that they all have favorable binding affinities for OCT1 as reflected ΔG scores. We predict from these analyses that vitamin K1 and olaparib are likely good binding ligands for OCT1.

**TABLE 3:** Comparison of the energies of the Ligand-OCT1 complexes.

Compound	ΔE_vdW	ΔE_ele	ΔE_GB	ΔE_SURF	G_gas	G_solv	Avg_ΔG
1-(m-phenoxyphenyl)-biguanide_OCT1	−45.2	−12.1	30.5	−2.3	−57.3	28.2	−29.1
1-(p-chlorophenethyl)-biguanide_OCT1	−48.7	−10.8	32.1	−2	−59.5	30.1	−29.4
2,3-Dihydro-1H-indene-2-yl acetate_OCT1	−42.3	−13.4	28.9	−2.7	−55.7	26.2	−29.5
Ethidium_OCT1	−50.1	−11.9	31	−2.4	−62	28.6	−33.4
Metformin_OCT1	−38.5	−14.2	27.8	−2.1	−52.7	25.7	−27
Sparteine_OCT1	−46	−12.5	29.4	−2.6	−58.5	26.8	−31.7
Vitamin_K1_OCT1	−43.9	−13.1	30	−2.5	−56.9	27.5	−29.4
Olaparib_OCT1	−44.8	−13	30.2	−2.2	−57.8	28	−29.8

Data were derived from Molecular Mechanics Poisson–Boltzmann Surface Area (MM-PBSA) calculations. Binding free energy was calculated as ΔG_bind = ΔE_vdW + ΔE_electrostatic + ΔG_polar + ΔG_nonpolar, where ΔE_vdW represents van der Waals interactions, ΔE_electrostatic represents Coulombic interactions, ΔG_polar represents polar solvation energy, and ΔG_nonpolar represents nonpolar solvation energy. All energy values are reported in kcal/mol.

### The total potential energy of the system reveals that vitamin K1 and olaparib exhibit energy properties similar to those of known OCT1 substrates

**FIGURE 3:**
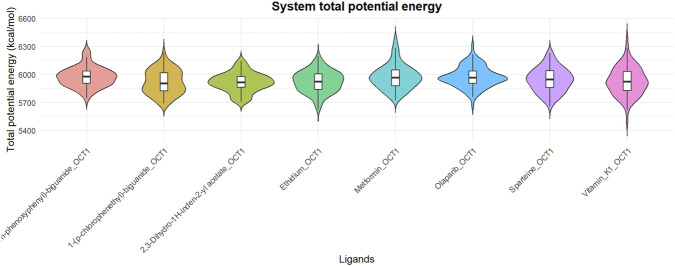
Illustrations of the distribution of the total potential energy for the indicated OCT1-ligands. Violin plot of the distribution of the total potential energy (kcal/mol) for the eight chosen ligands. The violin shapes illustrate the distribution density of energy values, with embedded box plots highlighting the median and interquartile ranges.

We next performed a violin plot analysis of the total potential energy distributions to determine the energetic landscape and dynamic stability of the ligand-bound OCT1 system during the molecular dynamic simulations. The plot compares the total potential energy profiles for the eight ligands interacting with OCT1. Each ligand is represented on the x-axis, while the y-axis shows the total potential energy (kcal/mol) (Figure 3), ranging from approximately 5700 to 6300. The violin shapes illustrate the distribution density of energy values, with embedded box plots highlighting the median and interquartile ranges (Figure 3). Ligands such as **1-(m-phenoxyphenyl)-biguanide and 1-(p-chlorophenethyl)-biguanide** showed tighter distributions, suggesting consistent potential energy, while all the other ligands, such as vitamin K1 and olaparib displayed longer spreads, indicating more variable interaction strengths. The fact that vitamin K1 and Olaparib behave similarly to a known high-affinity substrate, sparteine, with respect to total potential energy suggests that vitamin K1 and olaparib would also be suitable interacting ligands for OCT1.

### Metformin or vitamin K1 effectively competes for OCT1-dependent uptake of the high-affinity substrate ethidium bromide

**FIGURE 4:**
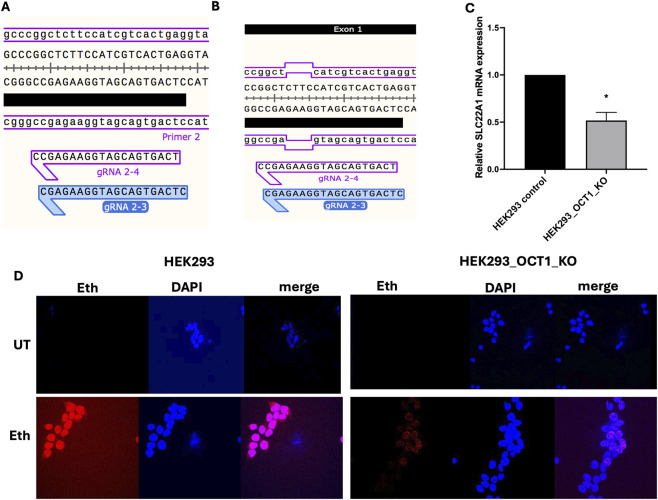
Verification of OCT1 deletion in HEK293 cells and ethidium bromide uptake by immunofluorescence staining. **(A, B)** Location of gRNA two to four targeting the SLC22A1 (OCT1) gene in Exon one and the deleted nucleotides revealed by Sanger sequencing, **(C)** OCT1 RNA expression level in the parent and the gene-deletion cells, and **(D)** Ethidium bromide (Eth) and DAPI staining in HEK293 control and HEK293 OCT1 KO cells. Briefly, HEK293 control and OCT1 deleted cells were plated on coverslips and treated with 10 µM ethidium bromide for 3 min. Cells were fixed, permeabilized, and nuclei were stained with DAPI. Imaging was performed on a Leica SP2 confocal microscope, and images were analyzed using ImageJ.

Molecular dynamics analyses identified energetically favorable and persistent ligand–OCT1 interactions, particularly for metformin and vitamin K1, suggesting that these compounds could competitively occupy the OCT1 transport pathway. We therefore experimentally assessed whether these ligands could interfere with OCT1-mediated uptake of the fluorescent substrate ethidium bromide, as an orthogonal validation of whether these predicted interactions translate into functional effects on OCT1-mediated transport. Ethidium was previously shown to be a high-affinity substrate for OCT1, with a K_m_ value of 0.8 ± 0.2 using the cell line that recombinantly expressed OCT1 (Haberkorn et al., 2021). Since ethidium bromide fluoresces with an excitation/emission of 300-360 nm/590-605 nm, it can be easily used to image its uptake into cells by confocal microscopy (Lee et al., 2009; Mihaljević et al., 2017; Pal et al., 1998). To do this, we monitor intracellular accumulation of ethidium bromide in the parent HEK293 cell line compared with a derivative HEK293 cell line deleted for the OCT1 transporter gene. The HEK293-OCT1 cell line deletion was generated by CRISPR and confirmed by Sanger DNA sequencing (Figures 4A,B). The OCT1-gene-deleted cells showed downregulation of OCT1 mRNA as determined by qPCR analysis (Figure 4C). These analyses confirm that the OCT1 gene was modified in the HEK293-OCT1-deleted cells, and this cell line was primarily used to confirm the uptake specificity of the OCT1 transporter for ethidium bromide.

The parent HEK293 cells incubated with 10 μM of ethidium bromide rapidly uptake the chemical compound within 3 mins (Figure 4D), and which accumulated in the nucleus, as it colocalized with DAPI staining of the nuclear DNA (Figure 4D). In contrast, a very weak uptake of ethidium bromide was observed in the HEK293-OCT1 deleted cells, consistent with the previous reports that OCT1 is required for mediating the high-affinity uptake of ethidium bromide (Figure 4D) (Lee et al., 2009; Mihaljević et al., 2017; Pal et al., 1998). The weak uptake of ethidium bromide in the HEK293-OCT1 deleted cells suggests the involvement of a second but minor transporter in the uptake of this chemical compound (Lee et al., 2009; Mihaljević et al., 2017; Pal et al., 1998).

**FIGURE 5:**
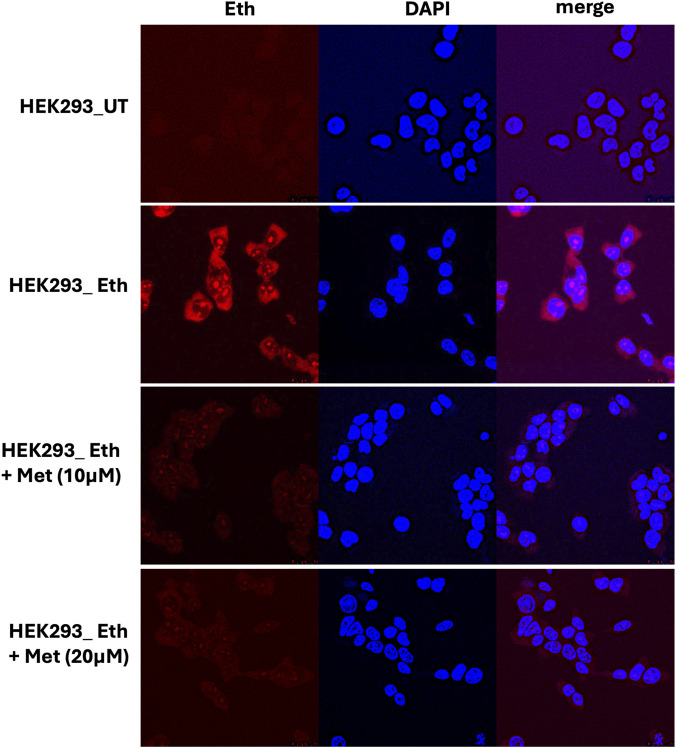
Metformin competes for ethidium bromide uptake into the parent HEK293 cells. Briefly, HEK293 control cells were plated on coverslips overnight, incubated with 10 µM ethidium bromide for 3 min, or with 10 µM ethidium bromide plus 10 or 20 µM metformin, delivered simultaneously for 3 min. After incubation, the cells were fixed, permeabilized, and stained with DAPI. Imaging was performed on a Leica SP2 confocal microscope, and the data were processed using ImageJ.

We initially investigated whether this imaging-based assay could be used to evaluate competitive interactions between ethidium bromide and the type 2 diabetic drug metformin, a well-characterized OCT1 substrate, during OCT1-mediated uptake (Shu et al., 2007; Dujic et al., 2014; Kawoosa et al., 2022). However, unlike ethidium bromide, metformin has a low affinity for OCT1 with a calculated K_m_ value of 1470 ± 190 using the recombinant cell line expressing OCT1 (Haberkorn et al., 2021). Surprisingly, metformin co-incubated with ethidium bromide, and both at the same concentration (10 μM), effectively blocked the uptake of ethidium bromide into the parent HEK293 cells (Figure 5). The confocal imaging analysis indicated that metformin might, instead, be a high-affinity substrate for OCT1, under the present experimental conditions that are different from the analysis obtained with the cell line recombinantly expressing OCT1 (Haberkorn et al., 2021; Zeng et al., 2023; Shu et al., 2007).

**FIGURE 6:**
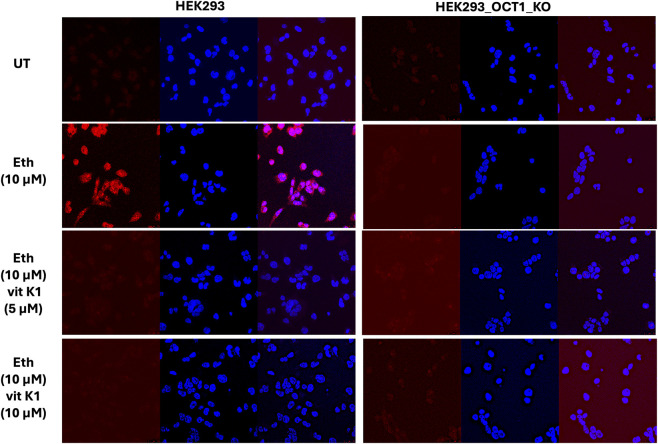
Vitamin K1 effectively blocks ethidium bromide uptake into the parent HEK293 cells. Briefly, HEK293 control and OCT1 deleted cells were plated on coverslips overnight. Cells were treated with 10 µM ethidium bromide only for 3 min, or treated with 10 µM ethidium bromide plus five or 10 µM vitamin K1 delivered simultaneously for 3 min. Following incubation, the cells were fixed, permeabilized, and stained with DAPI. Imaging was performed on a Leica SP2 confocal microscope, and analyses were processed using ImageJ.

Since the molecular dynamics simulations suggested that vitamin K1 may interact favorably with OCT1, we investigated whether vitamin K1 could compete with ethidium bromide for OCT1-mediated uptake using our confocal imaging assay. Co-incubation of 5 μM vitamin K1 with 10 μM of ethidium bromide for 3 min, followed by confocal microscopy imaging, resulted in a marked reduction in ethidium bromide uptake (Figure 6). This observation indicates that vitamin K1 effectively competes with ethidium bromide for OCT1-mediated transport activity, consistent with a model whereby vitamin K1 could function as a potent inhibitor of OCT1. Although inhibition through competition for uptake may be possible, other modes of inhibition cannot be excluded.

## Discussion

In this study, we employed docking and molecular dynamics simulations to investigate the behavior of a broad range of compounds that are reported to be transported by OCT1, as defined by uptake studies performed with cell lines recombinantly expressing OCT1 and by machine learning tools (Jensen et al., 2021; Haberkorn et al., 2021). At least 50% of the substrates can be docked onto OCT1 using computational tools. We selected a shortened list of 22 compounds for further analysis, based on binding energies ranging from −6.8 to −2.86 kcal/mol. In addition, five compounds that do not interact with OCT1 were included based on reported Km values, along with three potential OCT1 substrates, for a total of 30 compounds tested (Table 1). We subjected the 30 compounds to ligand topology filtering, which yielded a shorter list of eight ligands, including two of the three potential OCT1-interacting compounds, olaparib and vitamin K1.

The stable interactions observed by RMSD during molecular dynamics simulations focus on the importance of dynamic conformational changes (Haberkorn et al., 2021), solvent effects (Vidal-Limon et al., 2022), and ion-mediated stabilization (Pochini et al., 2024), such as sodium occupancy around Glu381 in OCT1. This stability suggests that docking and topology alone are insufficient to capture the full binding potential of certain substrates, as MDS reveal hidden binding modes facilitated by protein flexibility and Na^+^-dependent electrostatics (Halder and Das, 2022; de Angelo et al., 2025). On the contrary, when docking-predicted substrates fail to remain bound in MD simulations, docking and topology serve as critical filters to eliminate false positives and prioritize high-affinity candidates (Supplementary Figure S2). Thus, stable interactions in MD simulations despite poor docking emphasize the role of sodium and conformational adaptability in substrate recognition, while unstable interactions reinforce the utility of docking and topology in sorting substrates beyond *K*_*m*_ values (de Angelo et al., 2025). We acknowledge that a limitation of this study is the exclusion of an unambiguous lipid membrane, which may influence the conformational stability and dynamics of OCT1. Including a membrane environment in future molecular dynamics simulations will be important for better capturing physiologically relevant transporter behavior.

Molecular dynamics simulations revealed that the eight ligands form stable complexes with OCT1 via multiple interactions. For the ethidium-OCT1 complex, despite lacking hydrogen bonds and showing relatively low docking energy, the complex exhibits strong structural stability, as shown in Table 1 and Supplementary Figure S3. Based on these analyses, we showed by confocal microscopy that a low concentration of the fluorescent dye, ethidium bromide, rapidly entered and accumulated in the nucleus in the parent HEK293 cell line and only weakly into its derivative cell line deleted for the OCT1 gene by CRISPR, confirming previous reports that the high-affinity uptake of ethidium bromide into cells occurs primarily via the OCT1 transporter. The OCT1 gene-deletion cell line offers a unique tool as compared to previous studies using transiently transfected cell lines that overexpressed recombinant OCT1 or using expensive or unattainable whole animals lacking functional OCT1 (Lee et al., 2009; Mihaljević et al., 2017; Gorboulev et al., 2005). Based on our analysis, we considered ethidium bromide as a prototypical substrate for OCT1 as it is a planar cationic aromatic compound with a reported low *K*_*m*_ value (Gorboulev et al., 2005; Lee et al., 2009; Mihaljević et al., 2017). Therefore, we used the intensity of ethidium bromide accumulation into cells as a readout to monitor whether a compound has a comparable affinity and can compete for the uptake of this fluorescent dye. Together, the MD simulations and the *in vitro* data are complementary. MD simulations can identify potentially strong interacting ligands, while competition assays assess their functional impact on OCT1 transporter activity. It is noteworthy that ethidium bromide is limited to *in vitro* analysis and cannot be used for *in vivo* studies due to its carcinogenic properties.

Metformin is an established substrate for OCT1 reported to have a higher *K*_*m*_ value of 1470 µM for the transporter (Li et al., 2018), as compared to the lower *K*_*m*_ value (0.8 µM) for ethidium bromide. Metformin in complex with OCT1 exhibits strong structural stability, as in the case of ethidium bromide, suggesting that metformin could, in fact, be a high-affinity substrate for OCT1 (Shu et al., 2007). We demonstrated that the coincubation of metformin with the same low concentration as ethidium bromide (10 µM) efficiently competed for the uptake of the fluorescent dye, strongly indicating that metformin and ethidium bromide utilize the same saturable uptake transporter, OCT1, and that metformin exhibits at least a similar affinity for OCT1 as ethidium bromide. However, our observation is in contrast to the earlier findings that metformin is a low-affinity substrate defined by cell lines overexpressing OCT1 that could influence several parameters, as mentioned above, which differ from the experimental conditions herein (Zeng et al., 2023). We noted that our data do not provide a quantitative assessment of OCT1 transport kinetics for metformin, although metformin appears to have a comparable affinity for OCT1, similar to that of ethidium bromide. Nonetheless, assessing this properly will require determining the IC_50_ values or transport affinities for metformin versus ethidium bromide in a defined OCT1 expression system (e.g., OCT1-overexpressing or knockout cells) for rigorous kinetic analysis, but this is beyond the scope of the present study.

So far, there is no crystal structure of OCT1 or a co-crystal structure of OCT1-metformin, but a recent cryo-electron microscopy structure of OCT1 bound to each of four separate ligands including metformin, explains its promiscuous ability to recognize and transport different substrates (Zeng et al., 2023). OCT1 uses a general mechanism, as the Major Facilitator Superfamily of transporters, whereby the ligand is bound to the outward-facing state before entering an occluded state, followed by the inward-facing state that allows the release of the ligand into the cytosol. During these structural changes, as OCT1 moves exogenous compounds across the cell membrane, it mediates interactions with the ligands using charged, polar, and aromatic amino acid residues (Zeng et al., 2023). For example, it uses hydrophobic regions to stabilize the inward conformation and neutralize charges in the OCT1 binding pocket to facilitate the intracellular release of the ligand. The OCT1 inward binding pocket uses polar and aromatic residues to interact with thiamine, metformin, and fenoterol, suggesting a common mechanism for many of its promiscuous substrates (Meyer et al., 2020).

As mentioned above, the energy binding analysis of OCT1 substrate complexes interactions reveals that sparteine, 2,3-dihydro-1H-indene-2-yl acetate, and 1-(m-phenoxyphenyl)-biguanide exhibited lower radius fluctuations (Figure 2C; Table 2), suggesting dynamic changes toward compact conformations using Radius of Gyration although with weaker binding stability compared to consistently compact ligands such as vitamin K1 and olaparib (Figure 2C). Meanwhile, ethidium emerged as the strongest binding energy (Avg_ΔG −33.4 kcal/mol, Table 3) despite high atomic flexibility (Supplementary Figure S3), indicating that flexibility can enhance pocket fit when compensated by robust van der Waals and gas-phase interactions (G_gas −62 kcal/mol, Table 3) (Zhang et al., 2024). Even though metformin formed hydrogen bonds (Figure 2D), it showed the weakest binding energy (−27 kcal/mol, Table 3) due to high solvation costs (G_solv +25.7 kcal/mol) (see Table 3), and limited van der Waals contributions (ΔE_vdW −38.5 kcal/mol), yet metformin was effective at very low concentrations in competing for ethidium bromide uptake. These findings demonstrate that OCT1 affinity is driven more by nonpolar interactions and conformational adaptability than by hydrogen bonding alone (Suo et al., 2023), while other substrates, such as sparteine and ethidium, can maintain high-affinity for OCT1 by significantly balancing G_gas contributions against low solvation expenses (Pochini et al., 2024).

The reported OCT1 cryo-EM structures displayed the inward-open confirmation showing shifts in the C-terminal region of the transporter (Zeng et al., 2023; Zhang et al., 2024; Parker et al., 2021). In our molecular dynamics simulations, we identified the same amino acid residues Val37, Ile365, and Leu366, which Zeng et al., predicted to form a hydrophobic gate that prevents solvent from outside from penetrating between the N- and C-terminal bundles, as well as the Cys36 residue involved in forming an extracellular shield over the substrate (Zeng et al., 2023). We compared the cryo-EM data for the OCT1-thiamine-bound complex (Table 1) with the amino acid residues that interact with vitamin K1 identified in our docking studies, as shown in Supplementary Figures S1 and S2. Based on our analysis, we found that vitamin K1 contacts the same amino acid residues Cys36, Val37, Ile365, and Leu366, as reported for the cryo-EM for OCT1 bound to thiamine. Vitamin K1 also contacts OCT1 via the hydrophobic aromatic amino acid residues Phe249 and Tyr263, as well as the non-polar residues Gly152 and Gly261. The most striking observation is that the co-incubation of vitamin K1 with ethidium bromide completely abolished the uptake of ethidium bromide. Likewise, the co-incubation of the established substrate metformin with ethidium bromide also completely abolished the uptake of ethidium bromide, suggesting that vitamin K1 may share properties in common with the OCT1 substrates ethidium bromide and metformin. Moreover, MD simulations indicate that vitamin K1 and olaparib strongly interact with OCT1, owing to their compact conformations and defined binding energies, suggesting that olaparib and vitamin K1 may engage in a drug-drug interaction, competing for substrate uptake by OCT1. It is noteworthy that vitamin K1 at a two-fold lower concentration than ethidium bromide (5 µM versus 10 µM) effectively blocked the uptake of ethidium bromide, raising the possibility that vitamin K1 may possess a significantly higher affinity as a novel ligand for OCT1. Our current data show that vitamin K1 effectively blocked OCT1-mediated transport of ethidium bromide in a manner similar to metformin blocking the dye uptake by OCT1. While the evidence supports the notion that vitamin K1 is a potent inhibitor of OCT1-mediated transport of ethidium bromide, it cannot be excluded that vitamin K1 crosses the membrane via OCT1-mediated transport. Thus, resolving this perceived limitation of the work will require dedicated uptake studies in the future, for example, using radiolabeled or LC–MS-based assays. If vitamin K1 is confirmed to be a high-affinity substrate of OCT1, variants that alter OCT1 transport activity could affect vitamin K1 uptake and tissue distribution, potentially disrupting vitamin K1 homeostasis. Such alterations may impair the γ-carboxylation of vitamin K–dependent coagulation factors, including factor VII, thereby increasing the risk of defective blood coagulation and bleeding-related disorders (Schulte et al., 2014).

## Data Availability

The original contributions presented in the study are included in the article/Supplementary Material, further inquiries can be directed to the corresponding author.
