# Supplementary material for: Applying molecular dynamics simulations to validate OCT1 substrates uncovers vitamin K1 as a high-affinity ligand
Source: Front Pharmacol. 2026 Jun 19;17:1830280. doi: 10.3389/fphar.2026.1830280 (PMC13328187; doi:10.3389/fphar.2026.1830280)
Supplement: Supplementary file 1 [file Supplementaryfile1.docx]

| 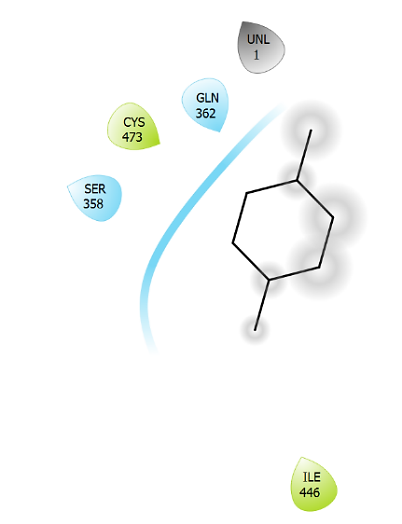 | 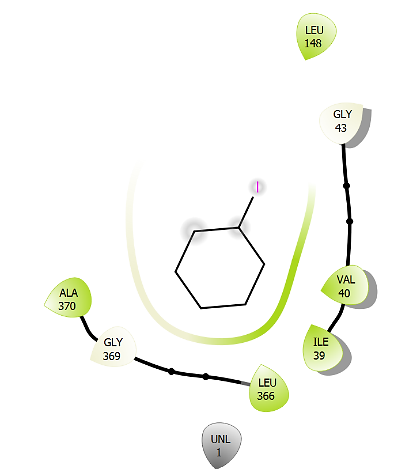 |
| --- | --- |
| 4.1-(m-phenoxyphenyl)-biguanide | 15. 2,3-Dihydro-1H-inden-2-yl acetate(2,3-dihydro-1H-inden-2-yl-biguanide) |
| 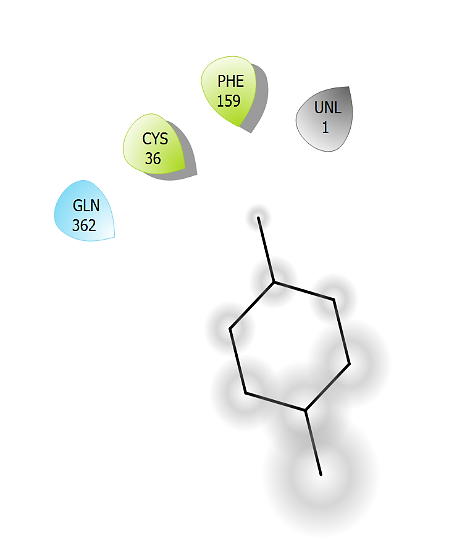 | 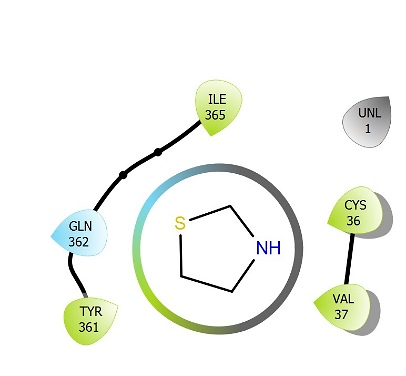 |
| 5.1-(p-chlorophenethyl)-biguanid | 39. Famotidine |
| 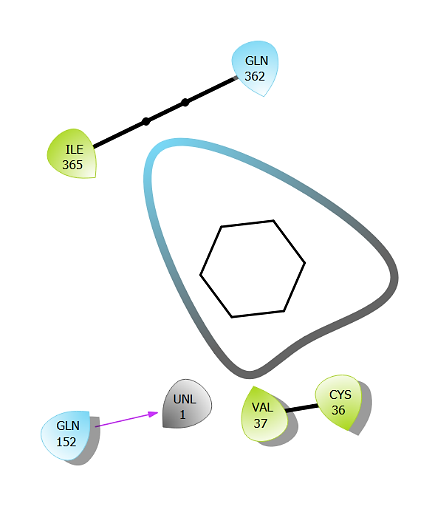 | 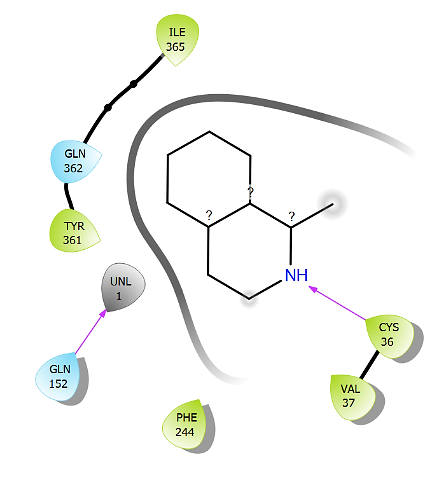 |
| Phenformin | Salbutamol-(R) |
| 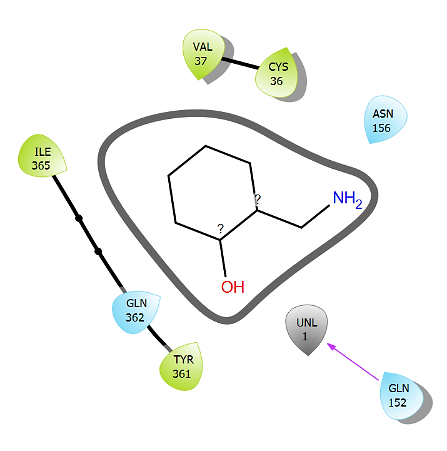 | 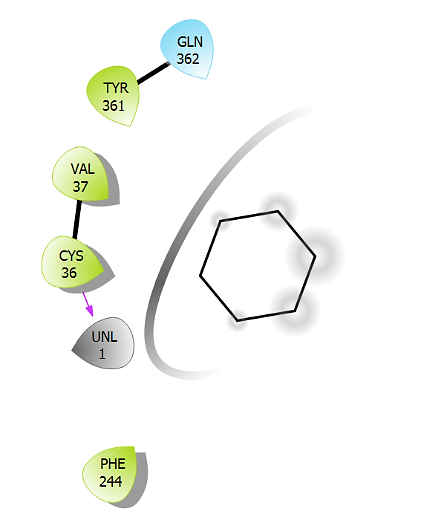 |
| Sparteine | Tropisetron |
| 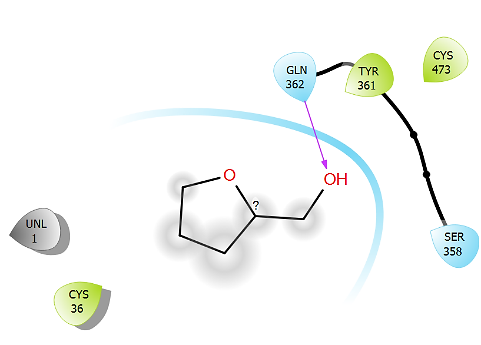 | 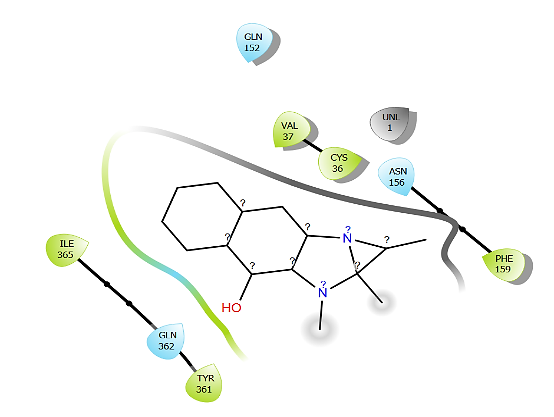 |
| Xamoterol (R) | YM155 |
| 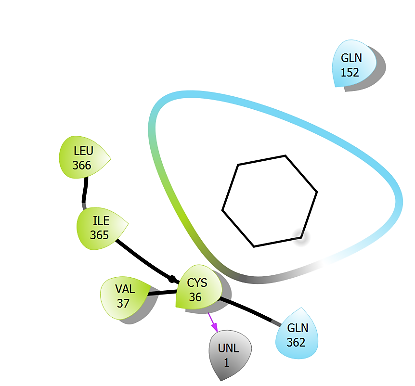 | 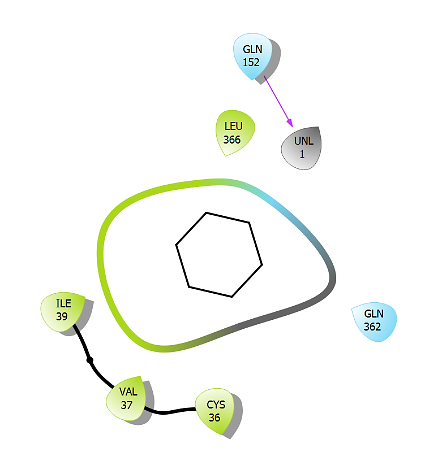 |
| Noroxycodone | Lamivudine |
| 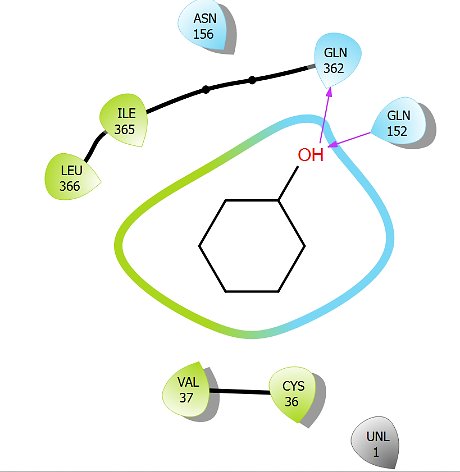 | 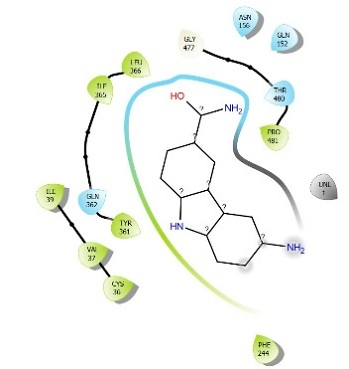 |
| Edrophonium | . Reproterol |
| 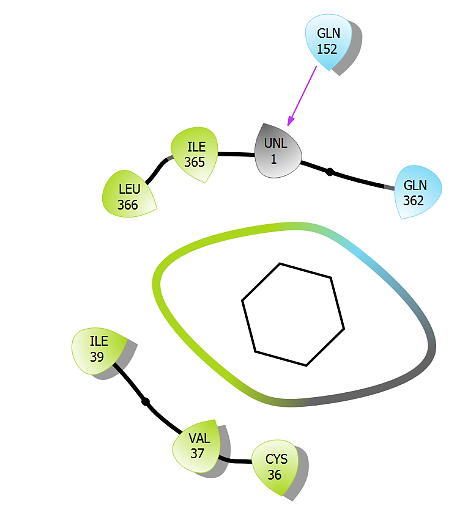 | 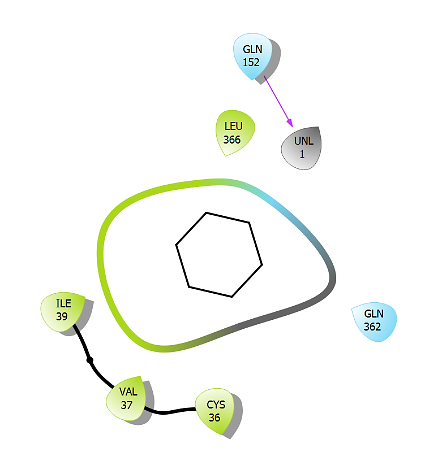 |
| Ethidium | Etilefrine-(S) |
| 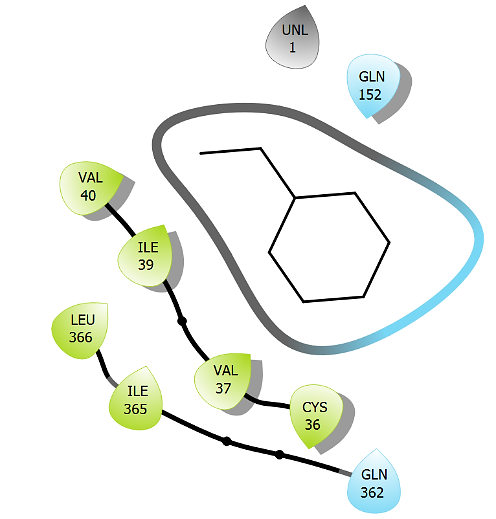 | 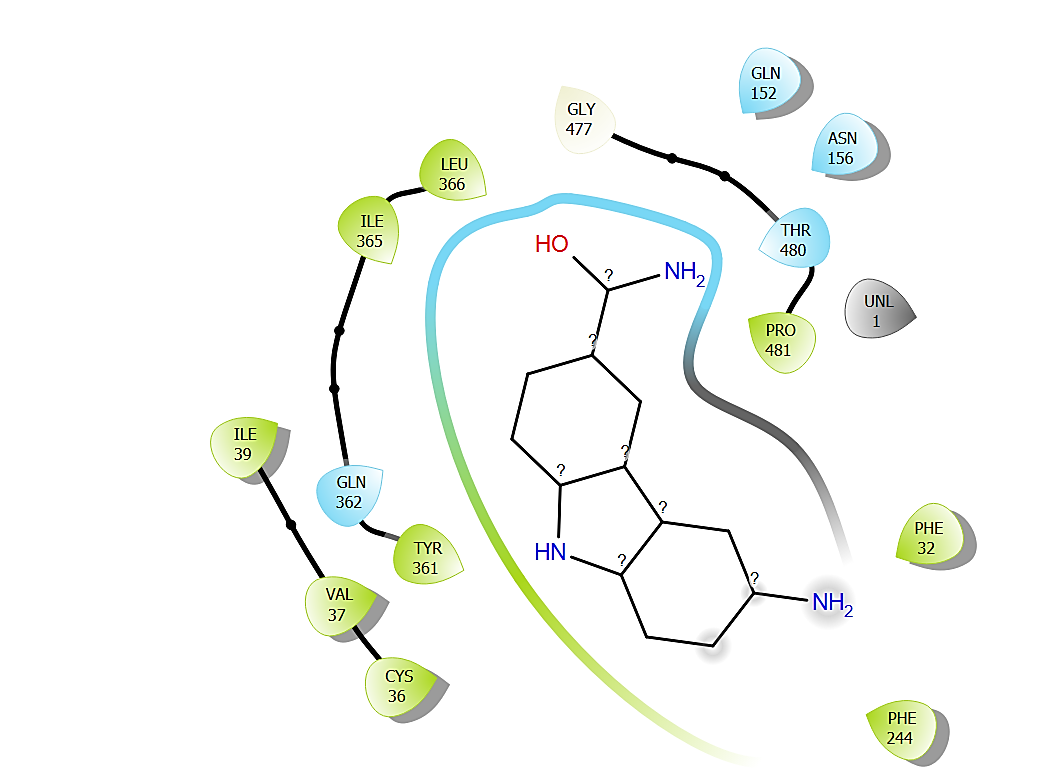 |
| Thiamine | serotonin |
| 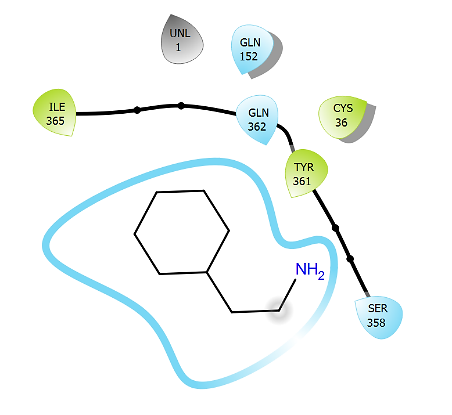 | 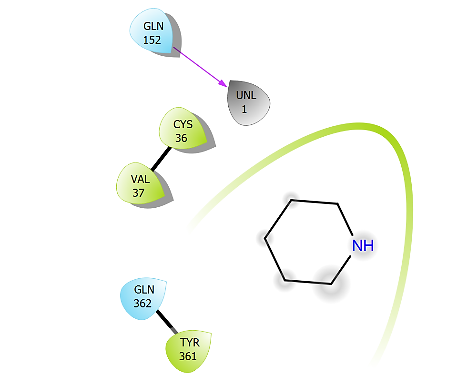 |
| Berberrubine | Morphine |
| 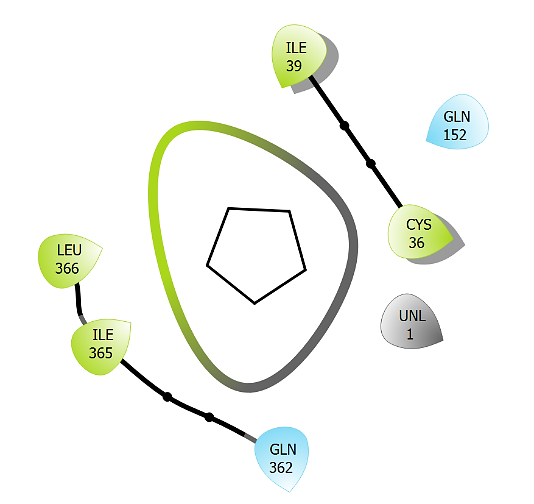 | 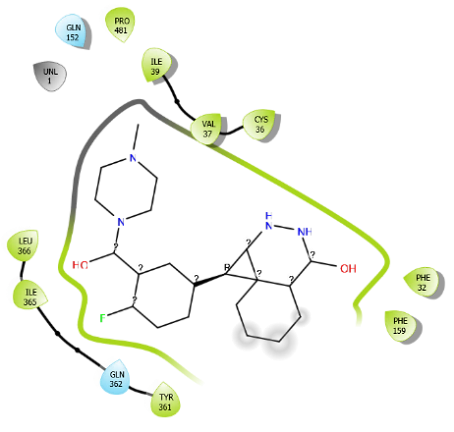 |
| Ganciclovir | Olaparib |
| 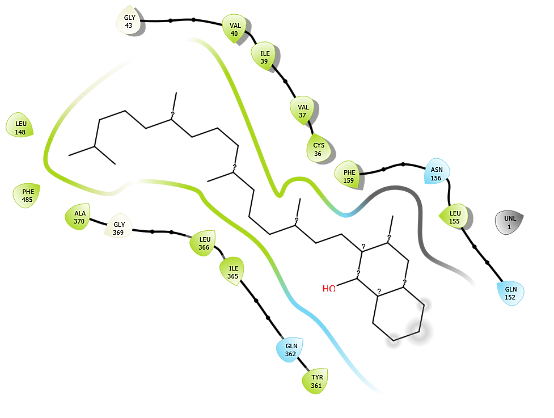 | 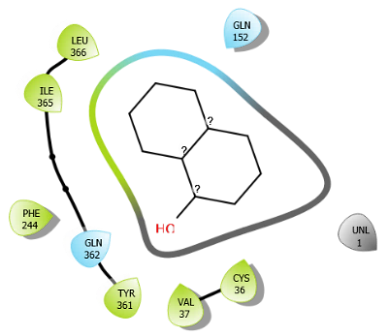 |
| Metformin | Vitamin K1 (Phytonadione |
| 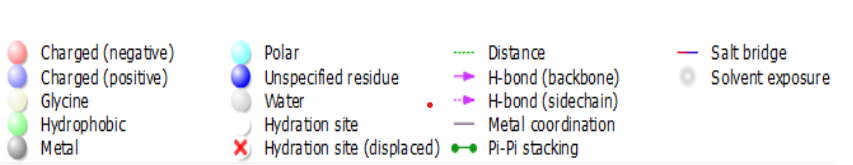 | |
| Figure S1: The 2D structures of docking interaction between the lower energy ligand-OCT1 receptor, as shown in the selected ligands table. The arrows indicate the hydrogen bond between the amino acids in the active site of the OCT1 receptor. The active amino acids of the polar atom, and of the hydrophobic atoms that surround the ligand, are shown in grey UNL. | |

| A | B |
| --- | --- |
| 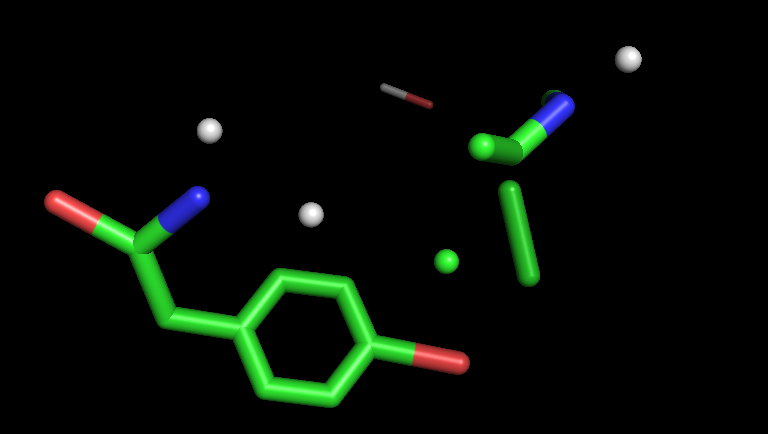 | 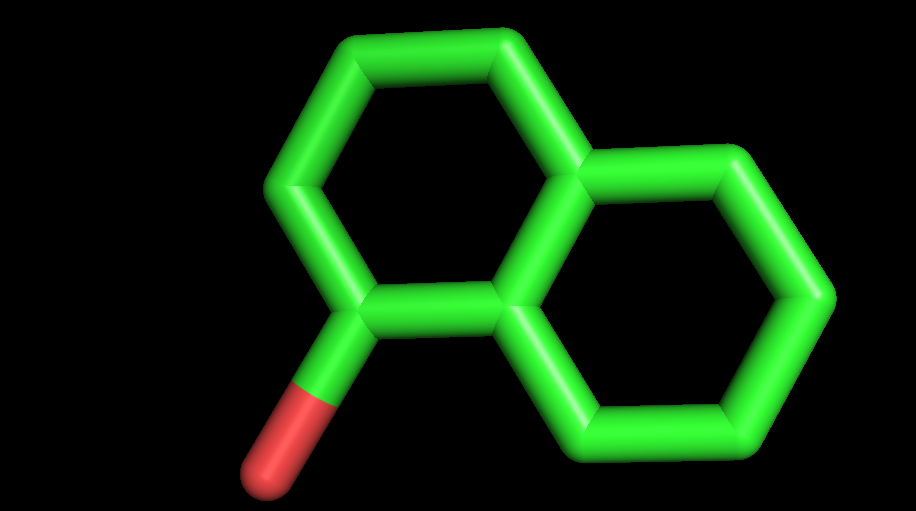 |
| Figure S2. Comparison of a compound that fails topology filtering versus a substrate that passes the filtering. (A) Atenolol fails topology filtering due to its linear polar structure, which lacks extended hydrophobic anchoring groups. (B) Vitamin K1 passes topology filtering because its long hydrophobic tail and aromatic ring system provide a favorable topology for OCT1 substrate recognition*.*  . | |

| 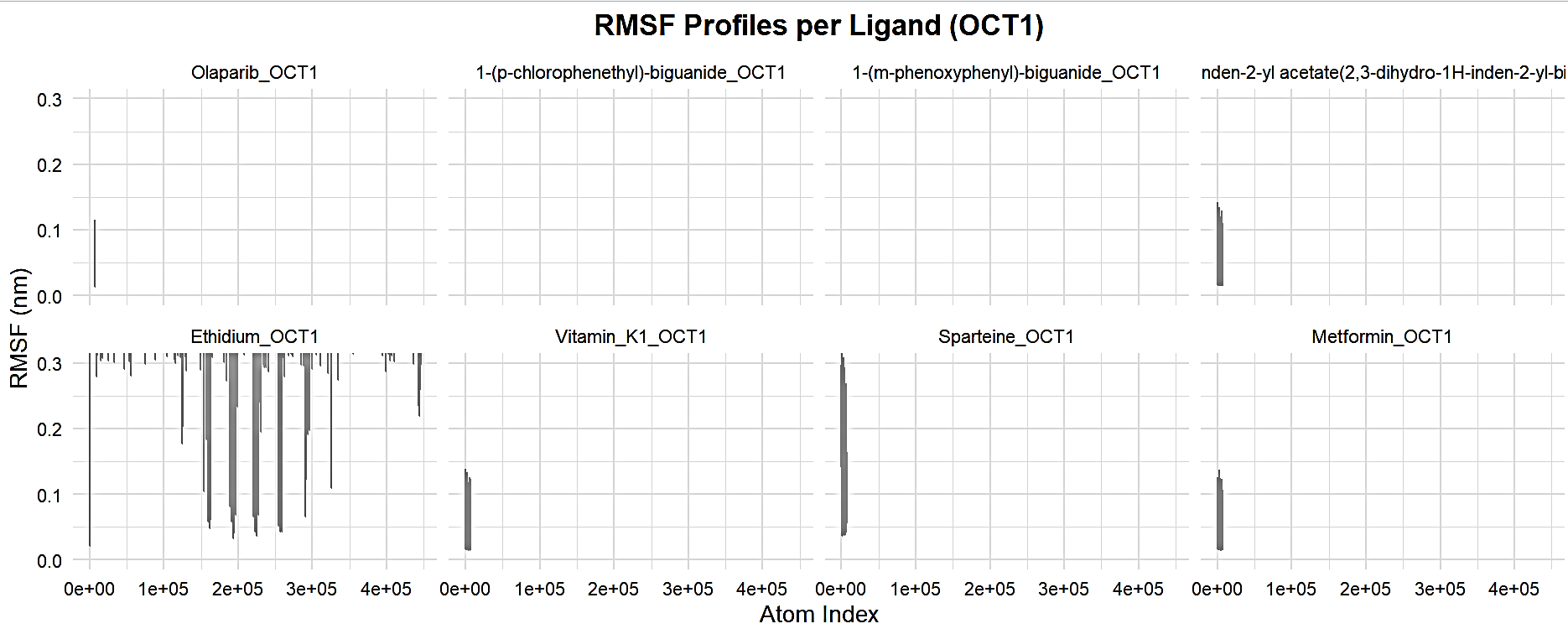 |
| --- |
| Figure S3(A). Root means square fluctuation (RMSF) patterns were used in molecular dynamics trajectory analysis for evaluating the OCT1 protein's flexibility at the residue level. To highlight atomic oscillations throughout the protein structure during simulation, grids were created in GROMACS using RMSF data from eight specific ligand-bound OCT1 complexes. |
| 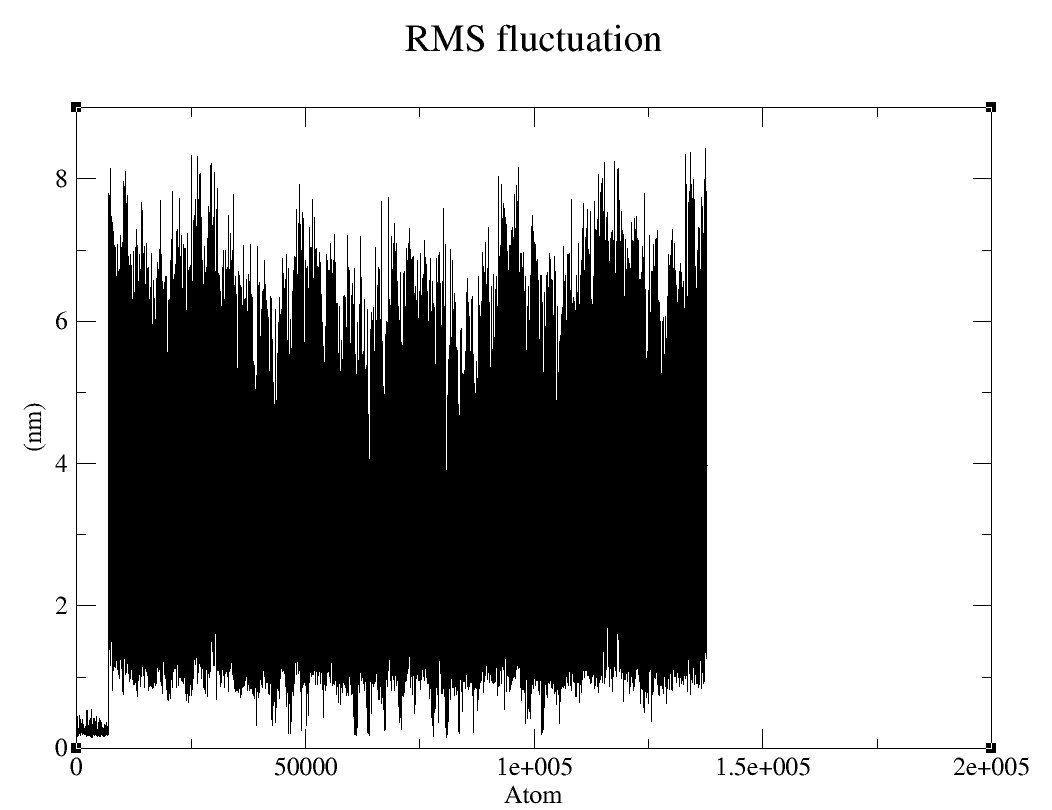 |
| S3(B): **RMSF of the apo form of OCT1**. RMSF of OCT1 alone showed residue-level values in a comparable modest range (~0.40–0.65 nm), indicating the natural flexibility of OCT1. |

| 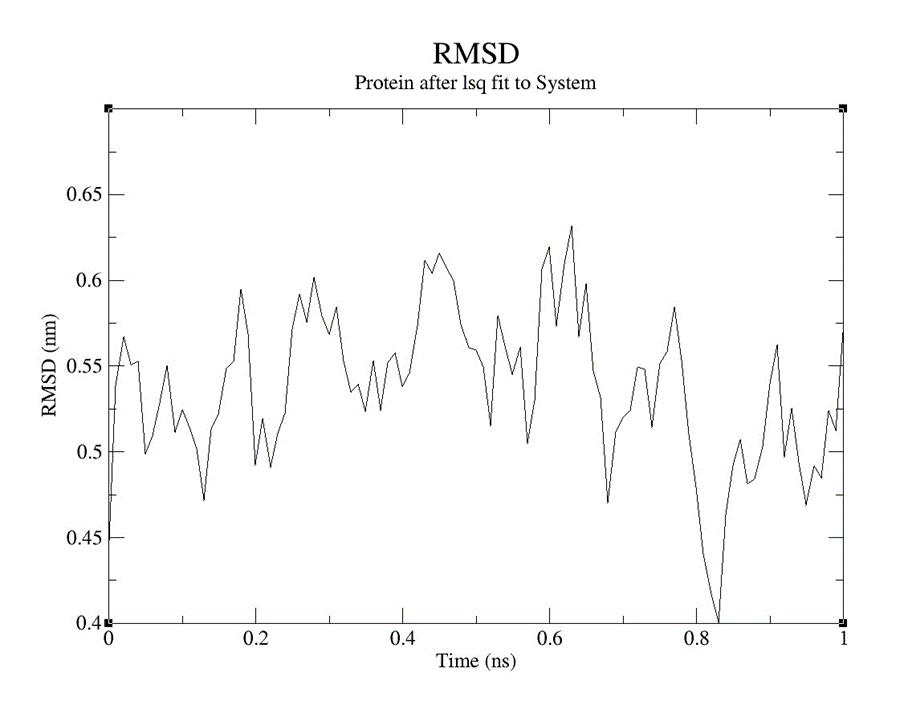 |
| --- |
| Fig. S4. RMSD analysis of the apo-OCT1 showing moderate backbone deviation values changing between 0.4 and 0.65 throughout the OCT1 simulation. |
